# Multiple Forms of Multifunctional Proteins in Health and Disease

**DOI:** 10.3389/fcell.2020.00451

**Published:** 2020-06-10

**Authors:** Adriana Espinosa-Cantú, Erika Cruz-Bonilla, Lianet Noda-Garcia, Alexander DeLuna

**Affiliations:** ^1^Unidad de Genómica Avanzada (Langebio), Centro de Investigación y de Estudios Avanzados, Guanajuato, Mexico; ^2^Department of Plant Pathology and Microbiology, Robert H. Smith Faculty of Agriculture, Food, and Environment, The Hebrew University of Jerusalem, Rehovot, Israel

**Keywords:** multidomain proteins, protein promiscuity, moonlighting proteins, gene ontology, pleiotropy, mechanisms of disease

## Abstract

Protein science has moved from a focus on individual molecules to an integrated perspective in which proteins emerge as dynamic players with multiple functions, rather than monofunctional specialists. Annotation of the full functional repertoire of proteins has impacted the fields of biochemistry and genetics, and will continue to influence basic and applied science questions – from the genotype-to-phenotype problem, to our understanding of human pathologies and drug design. In this review, we address the phenomena of pleiotropy, multidomain proteins, promiscuity, and protein moonlighting, providing examples of multitasking biomolecules that underlie specific mechanisms of human disease. In doing so, we place in context different types of multifunctional proteins, highlighting useful attributes for their systematic definition and classification in future research directions.

At the time when George Beadle and Edward Tatum proposed the one gene–one enzyme paradigm (Beadle and Tatum, 1941), the key and lock mechanism of substrate recognition in enzymes was used to explain how proteins accelerate chemical reactions. In the early days of molecular biology, enzymes and other proteins were thus seen as highly specific monofunctional molecules. This theoretical framework has influenced our functional annotations in biological systems: once a role is assigned to a given protein, other functions at any level are usually overlooked (McKay and Ashley, 2008). However, since the 1940s many different mechanisms of multifunctionality have been described.

Proteins with multiple functions can influence the evolution of organisms, as they contribute to complexity and robustness. For instance, in early forms of life and in organisms undergoing genome reduction, multifunctionality may increase the functional repertoire of a limited set of genes (Kelkar and Ochman, 2013; Ferla et al., 2017). In addition, multifunctional proteins may coordinate the crosstalk of dissimilar biological processes, which is specially relevant in organisms with large genomes and intricate metabolic and regulatory pathways (Flores and Gancedo, 2011; Irving et al., 2018). Thus, the presence of undetected multifunctionality is a confounding factor in functional association analyses based on guilt-by-association, genotype-phenotype maps, and homology prediction and annotation (Jeffery, 1999; Salathe et al., 2006; Gillis and Pavlidis, 2011; Espinosa-Cantu et al., 2018). Identification and understanding of protein multifunctionality is needed to grant a better understanding of living systems in health and disease. Indeed, multifunctional proteins are frequently determinants of virulence and disease (reviewed in Henderson and Martin, 2011; Jeffery, 2015, 2018b; Franco-Serrano et al., 2018c), as we will emphasize.

Other genetic mechanisms that are also known to contribute to the overall functional diversity of living systems are not herein addressed, such as alternative promoters or splicing in transcription, overlapping ORFs, alternative UTRs in translation, or post-translational modifications (Ayoubi and Van De Ven, 1996; Delaye et al., 2008; Kelemen et al., 2013; Fellner et al., 2015; Mayr, 2016). Here, we review alternative definitions of protein multifunctionality and provide examples of multifunctional proteins associated to human disease. We focus specifically on multifunctionality at the protein level.

## Naming and Classifying Multiple Forms of Protein Multifunctionality

**FIGURE 1:**
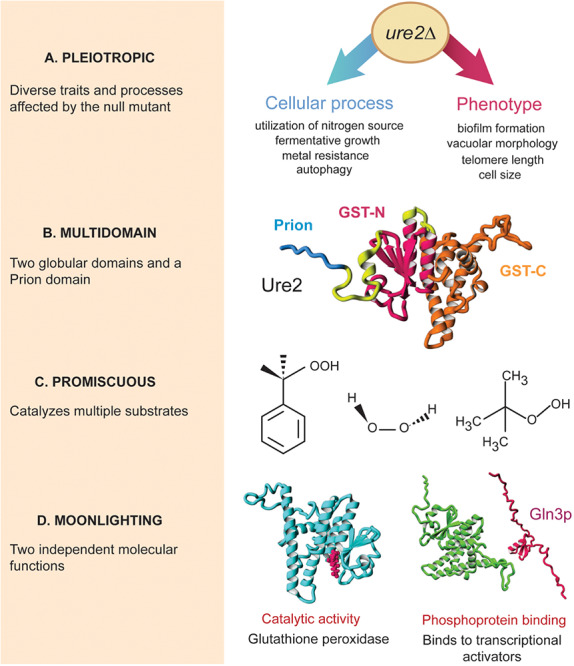
Different forms of protein multifunctionality in Ure2p. The Ure2 protein from budding yeast is a peroxidase enzyme that can form prions and is directly involved in nitrogen-catabolite repression by binding to and sequestering phosphorylated transcription factors in the cytoplasm, such as Gln3. **(A)** Deletion of *URE2* results in numerous phenotypes and observable traits related to different cellular pathways, which makes Ure2 a pleiotropic protein; only representative phenotypes associated to *ure2*Δ are shown. **(B)** Ure2 is a multidomain protein composed of an unstructured prion domain in addition to two globular domains typical of glutathione S-transferases (GST-N and GST-C). **(C)** The enzymatic activity of Ure2 is highly promiscuous: *in vitro*, Ure2 catalyze reactions with diverse substrates, involving different reaction mechanisms, within a single active site. Three different substrates for the glutathione-peroxidase activity of Ure2 are shown (from left to right: cumene hydroperoxide, hydrogen peroxide, and tert-butyl hydroperoxide). **(D)** The catalytic and binding activities of Ure2 are independent from one another: mutants lacking the Gln3-regulatory activity maintain their catalytic activity and such protein moonlighting is not explained by gene fusion, alternative splicing, or differential proteolysis. Protein images were generated using structure data from Protein Data Bank entry 1GgY, or Raptor X-predicted structures from Uniprot accessions P18494 and P23202.

To date, several non-mutually exclusive terms have been used to describe recurrent phenomena in which proteins perform two or more functions, notably pleiotropy, multidomain proteins, promiscuity, and moonlighting. Although the terms discussed here are widely used in the literature, there is an underlying lack of consensus in their usage, which may consequently hinder our understanding of the extent and biological significance of each of the different forms of protein multifunctionality. For instance, a single enzyme can be pleiotropic, multidomain, and show promiscuous and moonlighting activities (Figure 1; Aigle and Lacroute, 1975; Bai et al., 2004; Gancedo and Flores, 2008; Zhang and Perrett, 2009; The UniProt Consortium, 2018).

**FIGURE 2:**
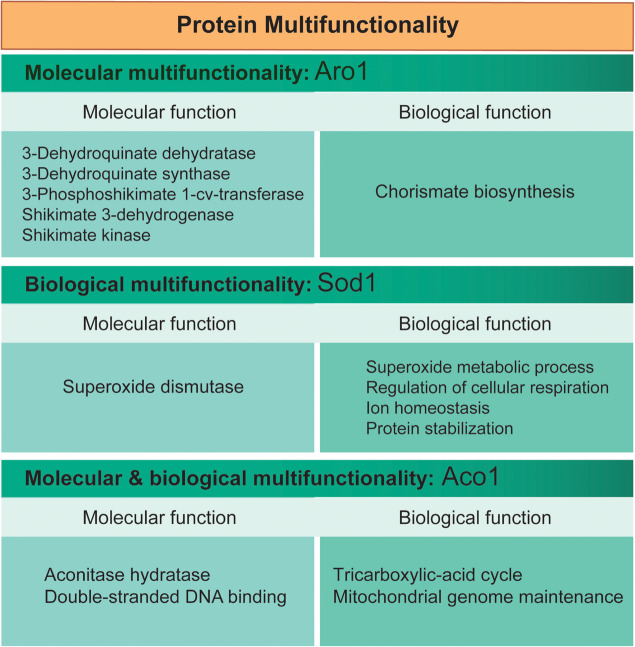
Different kinds of multifunctional proteins. Different kinds of multifunctional proteins may be described based on their “molecular” or “biological” functions. Examples are provided for three multifunctional proteins from *S. cerevisiae* (Aro1, Sod1, and Aco1); functional annotations (GO terms) are from the Saccharomyces Genome Database (www.yeastgenome.org).

The definition of multifunctionality fundamentally relies on the definition of protein function itself. Protein function is the potential activity with a given physiological role that a polypeptide with a single primary sequence can partake by interacting with other biomolecules or chemical compounds. Based on Gene Ontology (GO) annotations, protein multifunctionality may be described at different overlapping levels (Figure 2). The GO Consortium describes the function of a gene or gene product using three attributes: molecular function, biological process, and cellular component (Ashburner et al., 2000).

Pritykin et al. (2015) analyzed the genome-wide multifunctionality of proteins in yeast, fly, and humans. Between 20 and 26% of the characterized genes in these organisms are multifunctional proteins annotated in two or more significantly different GO biological process terms. A smaller percentage of proteins are annotated within two or more GO molecular function terms, but this observation can be partly explained by the fact that the number of annotations belonging to the molecular function classification is lower relative to the biological process class. Compared to other proteins in the restricted set of analyzed organisms, proteins annotated with more than one GO molecular function – which may include pleiotropic, multidomain, promiscuous, and moonlighting proteins – are typically longer in sequence, are frequently more broadly expressed, have more protein domains and higher fractions of disordered regions, and tend to be conserved and essential (Pritykin et al., 2015). Importantly, such multifunctional genes are more often involved in human disorders. These results highlight the convenience of a systematic classification of multifunctionality to uncover its prevalence, characteristics, and relevance in biological systems.

Diverse stimuli may trigger action of different functions of a multifunctional protein (reviewed in Jeffery, 1999, 2018b; Nobeli et al., 2009). Cellular conditions such as temperature, pH, concentration of metabolites, and presence or absence of ligands have been observed to regulate the functions of multitasking proteins. Differential expression, post-transcriptional modifications, intra- or extra-cellular localization, protein structural changes – from flexibility of small regions to complete refolding of an entire domain – and homo-or hetero oligomeric conformations are frequent molecular mechanisms of protein multifunctionality. Such mechanisms are not mutually exclusive; actually, they tend to take place concertedly in the occurrence and alternation of multiple functions.

## Pleiotropy May Reflect Multifunctionality at Different Levels

Pleiotropy refers to phenomena in which a single genetic factor contributes to different cellular, physiological, or organismic traits (Stearns, 2010; Paaby and Rockman, 2013; Zhang and Wagner, 2013). In genetic analysis, pleiotropy is assessed by describing the effects of mutations in measurable phenotypic traits. For example, the complete deletion of the *URE2* gene in the budding yeast *Saccharomyces cerevisiae* results in abnormal autophagy, decreased resistance to several cellular stresses, decreased telomere length, and increased cell size, among other phenotypes (Figure 1A). Likewise, mutations in Swc6, a chromatin-remodeling protein from *Arabidopsis thaliana*, result in different leaf, flower, and fruit morphologies (Lazaro et al., 2008).

The mutant phenotypes of a pleiotropic multifunctional protein may arise from the perturbation of a single molecular function that partakes in different biological processes, or multiple molecular functions, each with impact on a different biological process (Dudley et al., 2005; He and Zhang, 2006). A major challenge in genetics, cell biology, and biomedical research is to identify the molecular mechanisms underlying pleiotropy (van de Peppel and Holstege, 2005; He and Zhang, 2006). For example, the multiple phenotypes observed in phenylketonuria – behavioral and mental disorders along with specific physical traits – result from the inactivation of a single catalytic activity in the phenylalanine hydroxylase enzyme (reviewed in Hodgkin, 1998; Sarkissian and Gamez, 2005). Conversely, numerous non-enzymatic activities have been described for the pleiotropic enzyme glyceraldehyde-3-phosphate dehydrogenase, some involved in human pathologies including different types of cancer and Huntington’s, Parkinson’s, and Alzheimer’s diseases (reviewed in Tarze et al., 2007; Sirover, 2018).

## Multiple Protein Domains May Results in Manifold Functions

A protein domain is a polypeptide fragment with independent evolutionary history, structure, or function (Ekman et al., 2005). Multidomain proteins typically arise from gene fusion, whereby two protein fragments become linked together in a single open reading frame (Kirschner and Bisswanger, 1976). For example, in *Escherichia coli*, hydroxyethylthiazole kinase and thiamine-phosphate synthase are expressed in different proteins. However, in *S. cerevisiae* both catalytic activities are present in a single multidomain protein whereby each domain performs one enzymatic function (Jardine et al., 2002). The analysis of protein domain composition depends on the definition of domain and identification methods used, either based on protein-sequence comparison or structural properties (Ekman et al., 2005). For instance, the two domains of Ure2 are sometimes considered as subdomains of a single protein domain (Figure 1B), while the limits of each structural region vary frequently among protein databases. Importantly, not all multidomain proteins are multifunctional. The shikimate dehydrogenase from *E. coli* is a two-domain NAD(P)-oxidoreductase: while one domain binds the cofactor, the other couples with the substrate, in such a way that the two domains catalyze a single reaction in the aromatic amino-acid biosynthesis pathway (Michel et al., 2003).

An example of a multidomain protein involved in cardiovascular disease is the G-protein coupled receptor kinase 2 (GRK2, also known as beta-adrenergic receptor kinase). The role of GRK2 on the regulation of cardiac receptors is the result of successful coordination among the three protein domains that compose the enzyme: a regulator of G-protein signaling homology domain, a protein-kinase domain, and a pleckstrin-homology domain (Lodowski et al., 2003, 2005). While the disruption of the kinase gene can be lethal in mice, GRK2 overexpression has been linked to several cardiovascular diseases in humans (Ungerer et al., 1994; Akhter et al., 1999). Interestingly, the expression of the pleckstrin-homology domain in mice with GRK2 overexpression can revert the disease phenotype, and could therefore be used as a treatment strategy to improve compromised heart function (Akhter et al., 1999).

## Promiscuity Is (Or Is Not) True Protein Multifunctionality

Protein “promiscuity” describes a wide variety of related phenomena, all of which describe multiple catalytic activities or binding to multiple molecules (Hult and Berglund, 2007; Nobeli et al., 2009; Copley, 2015, 2017). The meaning and implications of promiscuity will depend on who one asks (Copley, 2015). From the evolutionary and biochemistry perspective, promiscuity usually involves physiologically irrelevant activities, by means of either inefficient or fortuitous activities in non-natural cellular contexts (Khersonsky et al., 2006; Copley, 2017). In other fields, however, promiscuity typically describes broad specificity or binding to multiple ligands, substrates, or other macromolecules with no consideration to the relevance of the activities (Copley, 2015). In the former scenario pertaining enzyme activity, promiscuity is not a form of molecular multifunctionality, while in the latter cases it actually is.

The use of the term promiscuity also depends on the type of activity being described: in non-enzymatic activities, promiscuity is used to describe binding to several different molecules in a single or multiple structural locations (Humphris and Kortemme, 2007; Patil et al., 2010; Schreiber and Keating, 2011). For instance, thioredoxin from *E. coli* or human Ras proteins have been described as promiscuous hub proteins given their many protein-protein interactions. While thioredoxin interactions take place in the same protein interface, Ras interactions occur in different binding sites (Humphris and Kortemme, 2007). For catalytic activities, promiscuity refers to a single active site – although not necessarily within the exact same residues – that either binds to a diverse set of substrates (substrate promiscuity or ambiguity) or catalyzes different chemical transformations (catalytic promiscuity) (Khersonsky et al., 2006; Nobeli et al., 2009; Khersonsky and Tawfik, 2010; Copley, 2015, 2017). For example, yeast Ure2 has glutathione peroxidase activity on different hydroperoxide molecules *in vitro* (Bai et al., 2004) and it can also oxidize a wide variety of substrates through alternative reaction mechanisms (Zhang and Perrett, 2009; Figure 1C).

The altered efficiency of enzymes catalyzing different substrates has been linked to health complications, for instance those having to do with perturbed metabolism of exogenous compounds. Such is the case of serum paraoxonase 1 (PON1), a glycoprotein with broad substrate specificity capable of hydrolyzing organophosphates, lactones, and aromatic esters (Costa et al., 2003). PON1 is involved in a variety of roles, from pesticide sensitivity to a protective role against atherosclerosis by hydrolyzing oxidized lipids and preventing their accumulation in artery walls. Frequent human polymorphic variants of PON1 show different catalytic efficiencies to alternative organophosphorus compounds and are related to different pesticide susceptibilities: while variant PON1_R__192_ hydrolyzes paraoxon faster than PON1_Q__192_, PON1_Q__192_ hydrolyzes diazoxon, sarin, and soman faster than the other isozyme (Davies et al., 1996). The altered hydrolysis efficiency toward other lipidic compounds may also explain why the PON1_R__192_ variant has been associated with cardiovascular disease (Aviram et al., 2000; Costa et al., 2003).

Dysfunction of catalytic-promiscuous enzymes can also influence Alzheimer’s disease and cancer incidence. The omega class gluthatione s-transferases GSTO1 and GSTO2 can carry out two different chemical reactions in the same active site: gluthatione-dependent thiol transfer and dehydroascorbate reduction (Board and Menon, 2016). The functions of GSTO1 and GSTO2 confer their role in oxidative stress and xenobiotic detoxification; glutathionylation prevents proteins from being oxidized and dehydroascorbate reduction takes part on the recycling pathway of the antioxidant ascorbic acid (Board and Menon, 2016; Kim et al., 2017). Altered function of these gluthatione transferases may underlie the physiological conditions of several diseases: a GSTO2 variant with low expression levels has been found to be associated with an increased risk of Alzheimer disease at older age, while increased GSTO1 levels have been linked to a number of cancers such as pancreatic, esophageal, colorectal, and ovarian cancer, among others (Allen et al., 2012; Board and Menon, 2016).

## Moonlighting Proteins Multitask in Disparate Functions

Protein “moonlighting” was first defined as the phenomenon whereby a polypeptide performs two or more molecular functions that are not the result of gene fusion, splice variants, or multiple proteolytic fragments (Jeffery, 1999). “Gene sharing” was originally coined to refer to the phenomena where a gene specialized in a function performed alternative biological roles. While this term is sometimes used as a protein-moonlighting synonym, both phenomena may actually differ in their mechanisms and implications (Copley, 2012). The concept of moonlighting protein has continued to expand in a less restrictive definition, and is nowadays also used to name proteins showing unrelated roles in the organisms, irrespective of their evolutionary history (Chapple et al., 2015; Jungblut et al., 2016; Khan and Kihara, 2016).

The molecular activities of a moonlighting protein are usually described as unrelated to each other and independently of the primary sequence (Huberts and van der Klei, 2010; Flores and Gancedo, 2011). Indeed, experimental evidence for the characterization of a moonlighting protein is mostly provided by mutants that are defective in one molecular function without affecting the others (Jeffery, 2009; Gancedo et al., 2016; Espinosa-Cantu et al., 2018). In the case of yeast Ure2, mutants without nitrogen-catabolite repression activity maintain their enzymatic capacities (Figure 1D). One of the first examples of a moonlighting protein was quite astonishing: the delta-crystallin of birds and reptiles is the exact same polypeptide as the argininosuccinate lyase enzyme; with its molecular function depending on the expression level and cellular context (Piatigorsky et al., 1988; Piatigorsky and Horwitz, 1996). In the lens, the protein is expressed at a high level and has a structural role, while in other tissues it is expressed at lower quantities and has an enzymatic function.

Moonlighting proteins with different molecular and functional characteristics have been described in a wide variety of organisms and the number of examples has increased in the last few years to almost 700 carefully curated moonlighting proteins (Hernández et al., 2014; Mani et al., 2015; Chen et al., 2018; Franco-Serrano et al., 2018b). Even though experimental setups have been aimed at revealing protein moonlighting in an unbiased manner (Su and Pillus, 2016; Espinosa-Cantu et al., 2018), still most known cases have been described by serendipity, either by studying a single protein in an exhaustive manner (e.g., the delta-crystalline Piatigorsky and Horwitz, 1996) or as rather surprising results from functional screens (Zelenaya-Troitskaya et al., 1995; Hall et al., 2004; Chen et al., 2005; Scott and Pillus, 2010; Scherrer et al., 2010; Chang et al., 2012).

Computational strategies have been developed to characterize moonlighting proteins and to identify novel candidates for further experimental confirmation. Alignment algorithms evaluating similarity to other proteins, functional domains, or motifs have been tested to predict the nature of alternative functions within a protein (Gomez et al., 2003). Protein-protein interaction databases can also reveal potential cases of protein moonlighting and provide suggestions to further test multifunctionality (Gomez et al., 2011). The topological properties of a protein-protein interaction network and GO annotations were used to identify proteins exhibiting signs of so-called “extreme multifunctionality” (Chapple et al., 2015). Khan and Kihara developed a method based on machine learning of different “omic” data, such as expression profiles, phylogenetic profiles, protein-protein interactions, genetic interactions and gene ontology (Khan and Kihara, 2016). More recently, a method based on text-mining was developed to identify moonlighting protein candidates within publications hinting at semantically different functions (Jain et al., 2018).

As the number of known moonlighting proteins has increased, efforts have been made to understand how these molecules evolve and to guide in looking for more such multifunctional biomolecules. One of the first observations was that moonlighting proteins are enriched in conserved, highly expressed, proteins; however, this could also represent a bias where highly expressed and conserved proteins are also more likely of being studied (Huberts and van der Klei, 2010). In addition to their expression patterns, sequence and structural properties may be related to protein moonlighting. For example, it has been proposed that moonlighting proteins could be enriched in proteins with well-packed, independent structural domains with little evolutionary trade-offs between the functions. Disordered regions could also facilitate the accumulation of functional mutations and shifts between activities (Tompa et al., 2005). Yet, a correlation between domain composition, disordered regions, and moonlighting has not been documented unambiguously (Hernandez et al., 2011; Amblee and Jeffery, 2015; Chapple et al., 2015). With regards to the interactions, candidate moonlighting proteins interact with more functionally diverse proteins and more connected to other moonlighting proteins (Khan et al., 2014; Chapple et al., 2015). Importantly, moonlighting proteins tend to be involved in mechanisms of disease (Chapple et al., 2015; Franco-Serrano et al., 2018c).

Diseases and pathologies related to moonlighting phenomena are being constantly described (Sriram et al., 2005; Jeffery, 2011; Zanzoni et al., 2015; Henderson et al., 2016; Franco-Serrano et al., 2018c). It has been estimated that up to 78% of candidate moonlighting proteins are associated to a human disease, in contrast to a 17.9% disease association for proteins in general (Franco-Serrano et al., 2018c). Among the diseases with the highest representation among moonlighting candidate proteins are those related to cancer (27%), immune system (17%), and nervous system (14%). As moonlighting proteins can have unrelated functions, it is expected that dysfunction of one or more of the protein functions gives rise to complex phenotypes. So far, moonlighting candidates have been found to be enriched in proteins that are involved in more than one disease and associated to complex instead of monogenic diseases (Zanzoni et al., 2015). These proteins are associated to phenotypically dissimilar diseases that share co-morbidity patterns, hinting this could result from the perturbation of different processes of a single moonlighting protein.

The essential enzyme glucose-6-phosphate isomerase (GPI) is an example of a moonlighting protein that has been linked to anemia and neurological pathologies due to its multifuncional nature. The dimeric form of GPI catalyzes the reversible conversion of fructose-6-phosphate to glucose-6-phosphate in glycolysis (Kugler and Lakomek, 2000), while the monomeric proteins show a neurotrophic function, independently of catalytic activity, which promotes the survival of embryonic spinal neurons, skeletal motor neurons, and sensory neurons (Gurney et al., 1986). Mutations in GPI cause a disorder known as GPI-deficiency, characterized by breakage of red blood cells (hemolytic anemia) due to impairment of glycolysis (Kugler and Lakomek, 2000). Most forms of GPI-deficiency are associated to mutations affecting only the isoenzyme activity, but severe forms of the disease, including neurological pathologies – muscular weakness and intellectual disability – are associated to mutations speculated to prevent correct protein folding disrupting both its catalytic and neurotrophic functions (Kugler et al., 1998).

Post-translational modifications involved in the modulation of moonlighting activities have been associated to the development of human disease. Phosphorylation, acetylation, and ubiquitination – among other protein modifications – affect the cellular localization and the binding affinity of proteins to other molecules (Jeffery, 2016; Zanzoni et al., 2019). For example, the S-nitrosylation of the glyceraldehyde 3-phosphate dehydrogenase (G3PDH) protein promotes the shift from the catalytic function to a protein-binding function (Hara et al., 2005). In this non-catalytic activity, G3PDH binds to a E3-ubiquitin ligase, Siah1, which promotes enter to the nucleus, where this protein complex initiates apoptosis. This signaling cascade has been proposed to take part in the neurodegeneration observed in Parkinson’s and Alzheimer’s diseases. Importantly, inhibition of S-nitrosylation of G3PDH by the drug L-deprenyl prevents cell death in cerebellar granule neurons in mice and delays the progression of Parkinson’s disease in humans (Parkinson Study Group, 1993; Hara et al., 2006).

Disease can also be triggered by a protein acquiring a novel activity due to a structural perturbation (mutation or conformational change), in which case the new function is known as “neomorphic moonlighting function” (Jeffery, 2011). For example, the beta-2-microglobulin acquires a new aggregation function besides its normal function in the major histocompatibility complex of presenting peptide antigens (Miyata et al., 1993; Jeffery, 2011). In the context of diminished kidney function or patients in long-term dialysis, beta-2-microglobulin is accumulated in the blood. This condition favors the conformational change to the amyloid fiber form and further protein aggregation, which ultimately leads to the development of amyloidosis characterized by arthropathy, lytic bone lesions, and chronic kidney disease.

Moonlighting proteins are not relevant to disease only due to their malfunction in humans, but also due to their role in pathogenesis and interaction with a human host (Henderson and Martin, 2011; Franco-Serrano et al., 2018a; Jeffery, 2018a). Intracellular proteins of pathogenic bacteria with previously described roles as metabolic enzymes (G3PDH), chaperones (Hsp60), or elongation factors (EF-Tu) have been found to exert a secondary function on the surface of bacterial cells as adhesins binding directly to host cells or extracellular matrix to initiate the infection process (Amblee and Jeffery, 2015; Jeffery, 2018a; Kopeckova et al., 2020). Moonlighting proteins can also influence virulence by providing antibiotic resistance to bacteria (Sengupta et al., 2008) and influencing nutrient uptake of the pathogen (Modun and Williams, 1999). Overall, 25% of known moonlighting proteins are classified as virulence factors and are found in some pathogens of interest such as *Staphylococcus aureus*, *Helicobacter pylori*, *Haemophilus influenza* and *Neisseria meningitides*, among others (Franco-Serrano et al., 2018a; Jeffery, 2018a).

## Toward a Systematic Description of Protein Multifunctionality

Huge amounts of data are being produced from experimental and computational analysis in functional genomics and systems biology, making it urgent to unify definitions of protein multifunctionality in a more systematic manner. Recent advances in large-scale genetic and genetic-interaction screens (Bellay et al., 2011; Martin et al., 2015; Espinosa-Cantu et al., 2018), proteomics and protein-protein interaction databases (Gomez et al., 2011; Becker et al., 2012; Chapple et al., 2015; Wang and Jeffery, 2016), and metabolite screens (Sevin et al., 2017; Piazza et al., 2018), along with computational analyses of functional data (Ekman et al., 2005; Nam et al., 2012; Pritykin et al., 2015) have huge potential to reveal patterns of protein multifunctionality in living systems.

In this context, we note that certain features are good descriptors of the types of protein multifunctionality. Structural similarity, biological relatedness, structural autonomy, and physiological relevance of the different functions of a single protein will certainly influence the evolutionary scenarios and outcomes involved in the gain and loss of multifunctionality. For example, the lesser the structural autonomy between the activities in a protein, one would expect evolution of more similar molecular functions or the appearance of stronger evolutionary trade-offs. Duplication and divergence of the gene copies of multifunctional proteins with evolutionary trade-offs could result in single monofunctional proteins (Piatigorsky, 2007; Espinosa-Cantú et al., 2015). Unrelated biological functions may be more prone to evolve separately if they are antagonistic between each other. Therefore, it is possible that less structural autonomous functions would have more biological coherence. Functions with strong – or relevant – phenotypic effects will typically be subjected to strong selective pressures. Therefore, in proteins from mild to highly structurally dependent and relevant activities, one would expect co-evolution of functions. In contrast, those cases in which only one of the functions is under strong selection may be prone to mild or little structural autonomy, allowing mutations of the residues involved in the activity.

Pleiotropy, multi-domain proteins, promiscuity, and moonlighting are different biological phenomena related to protein multifunctionality. The description of different recurrent phenomena has been – and will certainly continue to be – useful to the understanding of protein multifunctionality. However, as often seen in biology, huge diversity in the mechanisms and related phenomena will most likely exist. Moreover, the terms and limits we assign to each phenomenon are also prone to evolve, thus developing a natural lack of consensus. Therefore, it is possible that an alternative, straightforward, annotation of protein multifunctionality may enable future endeavors to comprehend the diversity, extent, and significance of protein multifunctionality in a more systematic manner.

## Author Contributions

AE-C, EC-B, LN-G, and AD wrote the manuscript, read, and approved the final manuscript.

## Conflict of Interest

The authors declare that the research was conducted in the absence of any commercial or financial relationships that could be construed as a potential conflict of interest.
